# Preliminary Outcomes of a Five-Session Motivation-Centered Program for Internet Gaming Disorder: A Completer Analysis of a Randomized Trial

**DOI:** 10.3390/bs16091500

**Published:** 2026-08-26

**Authors:** Ruoyu Zhou, Nobuaki Morita, Yasukazu Ogai, Yoshiki Koga, Fan Yang, Wenjie Yang, Chunmu Zhu

**Affiliations:** 1 School of Future Education, Qingdao Hengxing University of Science and Technology, Qingdao 266100, China; zhouruoyu@qdhxu.edu.cn; 2 Faculty of Medicine, University of Tsukuba, Tsukuba 305-8577, Japan; nobuakim@nifty.com (N.M.); ogai.ys@md.tsukuba.ac.jp (Y.O.); 3 National Hospital Organization Kurihama Medical and Addiction Center, Yokosuka 239-0841, Japan; koga.yoshiki.mj@mail.hosp.go.jp; 4 Faculty of Letters, Arts and Sciences, Waseda University, Tokyo 162-8644, Japan; fan.yang@ruri.waseda.jp; 5 The Mental Health Center, Yunnan University, Kunming 650091, China; 6 Graduate School of Medicine, Tohoku University, Sendai 980-8575, Japan; zhu.chunmu.p1@dc.tohoku.ac.jp

**Keywords:** internet gaming disorder, behavioral addiction, university students, psychological intervention, gaming motivation, randomized controlled trial, China

## Abstract

Brief campus-based interventions for university students with elevated internet gaming disorder (IGD) symptoms have received little study. This randomized trial evaluated a five-session group program focused on gaming motives, self-regulation, and alternative sources of competence and self-worth. Sixty-three adult students at a Chinese vocational college who scored at least 20 on an adapted Gaming Disorder Scale for Adolescents were randomized to the program (*n* = 31) or an information-only control condition (*n* = 32). Assessments were scheduled at baseline, immediately after the intervention, and one month later. Because post-randomization outcome data were unavailable for 19 participants, the comparative analyses were restricted to 44 completers. The originally planned baseline-adjusted ANCOVA showed a lower adjusted T1 IGD score in the intervention group than in the control group (adjusted difference = −4.62, 95% CI −7.33 to −1.91, *p* = 0.0013). In a post hoc linear mixed-effects model, the between-group difference in change from baseline to post-intervention was −5.16 points (95% CI −7.81 to −2.51; Hedges g = −1.03). At one month, the difference was smaller and not statistically significant (−1.67 points, 95% CI −4.31 to 0.98). None of the secondary outcomes showed a significant interaction. Overall attrition was 30.2%. These preliminary findings concern selected completers rather than an intention-to-treat effect, and the between-group difference was not evident at one month. Retrospective registration, unavailable outcomes for non-completers, and post hoc analyses limit the strength of the inference. The trial was retrospectively registered in UMIN-CTR (UMIN000055209) on 9 August 2024.

## 1. Introduction

Internet gaming disorder (IGD) is listed in the DSM-5 as a condition for further study, while the ICD-11 recognizes gaming disorder as a mental and behavioral disorder characterized by impaired control over gaming, increasing priority given to gaming, and continued play despite harmful consequences (American Psychiatric Association, 2013; Reed et al., 2019). The two frameworks overlap but are not interchangeable, and prevalence estimates depend heavily on the instrument, population, and diagnostic threshold (Fam, 2018; Long et al., 2018; Pontes et al., 2021; Zhou et al., 2024a). A recent umbrella review reported a median IGD prevalence of 6.20% across meta-analyses (interquartile range, 4.25–8.30%), while also documenting substantial variation by diagnostic criteria, assessment tool, population, and region (Poon et al., 2026). This estimate should not be interpreted as the prevalence specifically among Chinese university students. University students may be particularly susceptible because academic pressure, changing social roles, and relatively unstructured leisure time can make gaming an accessible source of relief, identity, competence, and social approval (China Internet Network Information Center, 2022; Gu & Mao, 2023; Lei et al., 2016). More severe IGD symptoms have been associated with psychological distress, sleep and attention problems, strained relationships, and poorer academic functioning (Mihara & Higuchi, 2017; Petry & O’Brien, 2013).

The Interaction of Person-Affect-Cognition-Execution model proposes that addictive online behavior develops through the interaction of personal vulnerabilities, affective and cognitive responses, motivational expectancies, and executive control (Brand et al., 2019). In this account, the reason for gaming is as important as the amount of time spent gaming. Playing for enjoyment or skill development is not equivalent to playing to escape distress, satisfy external demands, or compensate for an unstable sense of identity (Bäcklund et al., 2022; Ballabio et al., 2017). Accordingly, an intervention may need to address maladaptive gaming expectancies and motives, strengthen self-regulation, and help students meet needs for competence, identity, and social recognition outside gaming.

Psychological treatment research provides a basis for intervening but does not yet identify a single well-established format. Cognitive behavioral therapy has been studied most extensively and can reduce IGD symptoms, while systematic reviews emphasize variation in intervention content, diagnostic definitions, comparison conditions, and follow-up, as well as small samples and risk of bias in many trials (Y. Chen et al., 2023; King et al., 2017; Stevens et al., 2019). Craving-focused, mindfulness-informed, group-based, and internet-delivered programs have also reported favorable outcomes (Deng et al., 2017; Su et al., 2011; Yao et al., 2017; Zajac et al., 2017). Thus, the question is not whether any psychological approach can help, but which targets, dose, and delivery format are feasible and sufficiently durable for a particular setting.

Existing interventions illustrate a wide range of intensity. In outpatient settings, STICA combined 15 weekly group sessions with up to eight individual sessions (Wölfling et al., 2019), whereas an eight-week group-based Mindfulness-Oriented Recovery Enhancement trial in U.S. adults reported improvements in IGD criteria, craving, and maladaptive gaming cognitions through three-month follow-up (Li et al., 2017). At the briefer end, the school-based PROTECT program delivered four 90-min cognitive behavioral group sessions to at-risk German adolescents and found greater symptom reduction over 12 months than assessment only (Lindenberg et al., 2022). These studies support structured psychological work but also show that findings from clinical adults, school prevention, and different treatment doses cannot be assumed to transfer directly to a Chinese tertiary-college service. A randomized e-health trial among Indian college students further suggested that a low-intensity intervention could improve gaming-related knowledge and self-regulation skills (Balhara et al., 2023). However, a recent umbrella review concluded that the overall certainty of evidence for digital-addiction interventions remains limited, supporting further rigorous evaluation of scalable campus-based approaches (Lu et al., 2025).

The closest contextual comparator is Ji and Wong’s (2023) eight-session integrated motivational cognitive-behavioral group intervention for adolescents in a Chinese secondary vocational school. That randomized waiting-list trial used intention-to-treat analyses, examined motivational mechanisms, and assessed three- and six-month outcomes. It established an important benchmark for motivational group work in Chinese vocational education, but it did not answer whether a shorter program is workable for adult students in tertiary vocational education. Evidence also remains limited on interventions that explicitly connect gaming motives and self-control with alternative sources of competence, self-worth, and action outside gaming. The present study therefore addresses a narrower development-stage gap: the immediate and one-month outcomes of a five-session, motivation-centered campus program among selected completers, while treating durability and implementation at scale as questions for future trials.

The present program was developed from two earlier studies within the same doctoral project. The first linked lower self-control with more severe IGD symptoms and found that internet-use motives partly accounted for this association among Chinese university students (Zhou et al., 2024b). The second used regression-tree analysis to identify combinations of introjected regulation, low self-control, psychological distress, identity-related motives, self-esteem, and competence-related motives in students with higher IGD scores (Zhou et al., 2025). These findings shifted the intervention away from a simple focus on gaming time and toward the functions gaming served, dependence on external evaluation, and opportunities to build competence and self-worth outside gaming.

We therefore evaluated a five-session group program combining psychoeducation, self-distancing writing, cooperative problem solving, mindfulness practice, and behavioral planning. Five sessions were chosen because this was the shortest sequence that retained the planned progression from recognizing problematic patterns, to examining gaming functions and building competence outside gaming, and finally to practicing self-regulation and planning change. The format could be completed within the college teaching calendar; it was not intended to define an optimal treatment dose or to replace more intensive clinical care. The originally planned primary question was whether baseline-adjusted IGD symptoms differed between groups immediately after the program. Post hoc analyses further described changes through the one-month follow-up. Daily internet use, psychological distress, self-esteem, self-control, readiness to change, and selected gaming motives were examined as exploratory outcomes.

## 2. Materials and Methods

### 2.1. Trial Design, Reporting, and Registration

This single-center, two-group parallel trial used a 1:1 allocation ratio. Assessments were completed at baseline (T0), immediately after the intervention period (T1), and one month after T1 (T2). Reporting follows CONSORT 2025 (Hopewell et al., 2025).

Recruitment began on 10 June 2024, and the trial was entered in the UMIN Clinical Trials Registry on 9 August 2024 (UMIN000055209; https://center6.umin.ac.jp/cgi-open-bin/icdr_e/ctr_view.cgi?recptno=R000063085, accessed on 24 August 2026). Registration was therefore retrospective. The delay arose because the research team did not initially understand that prospective registration also applied to a non-pharmacological behavioral intervention delivered in a university setting. The eligibility criteria, intervention content, outcome measures, and assessment schedule had already been specified in the approved doctoral research protocol. The registry record has since been updated with the actual enrollment, participant flow, and study results.

The full protocol and statistical analysis plan were not publicly posted before recruitment. The University of Tsukuba repository contains the dissertation record and abstract, and the protocol materials are available from the corresponding author on request. The eligibility criteria, intervention content, assessment schedule, and IGD outcome were not changed after recruitment began. The mixed-effects models, effect-size estimates, attrition comparisons, and multiplicity correction reported in this article were added after data collection and are identified as post hoc. The doctoral thesis originally reported a baseline-adjusted ANCOVA at T1 and separate repeated-measures analyses within each group.

### 2.2. Setting and Timeline

The trial was conducted at Yunnan Land and Resources Vocational College in Kunming, China. Recruitment took place in June 2024 through campus posters, and students contacted the research team voluntarily. The five sessions were delivered over about three weeks during a four-week study period in June and July. T1 followed the final session, and T2 was completed about one month later, in August 2024.

### 2.3. Participants

Recruitment used a self-selected, criterion-based non-probability sampling approach: students responded voluntarily to campus posters and were then screened against prespecified eligibility criteria. Students were eligible if they were at least 18 years old, were currently enrolled at the college, scored 20 or higher on the adapted Gaming Disorder Scale for Adolescents (GADIS-A), understood the study, and gave written informed consent. The threshold was used for risk screening and did not represent a clinical diagnosis. Students were excluded if they were receiving treatment for a psychiatric disorder or had severe cognitive impairment or a major physical illness that would prevent participation. The 44 completers had a mean age of 19.66 years and included 16 men and 28 women.

Intervention participants were classified as completers if they attended at least four of the five sessions and completed all three assessments. Control participants entered the completer analysis if they completed all three assessments and their questionnaire data met the quality criterion. Thus, inclusion depended on post-randomization behavior and was not defined identically across conditions. The available dataset contained only the 44 completers. Individual baseline and post-randomization outcome records for the other 19 randomized participants were not available for analysis; the results are consequently interpreted as an exploratory comparison among selected completers.

### 2.4. Sample Size

The doctoral protocol used G*Power version 3.1.9.7 to plan a two-group, three-time-point repeated-measures design. Assuming a medium effect size (f = 0.25), a two-sided alpha of 0.05, and 95% power, the calculation indicated that 44 completers would be required (Cohen, 1988). Allowing for 20% attrition increased the minimum recruitment figure to 55, and an initial target of 80 was set to provide additional allowance for attrition and recruitment uncertainty. Actual recruitment was constrained by the college teaching schedule and the fixed implementation window. Because the intervention required several group sessions to be completed within the same academic term, recruitment ended after 65 students had been screened and 63 eligible students randomized. The randomized sample was below the initial target of 80, which may have reduced precision and power for smaller secondary and exploratory effects; the 44 completers equaled the number in the original primary calculation. The retained planning records do not contain every G*Power input, including the assumed repeated-measures correlation and nonsphericity correction. The calculation is therefore presented as a planning reference rather than as a fully specified confirmatory power analysis.

### 2.5. Randomization, Allocation Concealment, and Blinding

After eligibility, consent, and baseline assessment, participants were assigned in a 1:1 ratio using a computer-generated sequence created in R version 4.4.0 by a researcher who was not involved in screening or intervention delivery. The records indicate simple randomization, with no blocking or stratification. Staff responsible for enrollment did not have access to the sequence before assignment, and allocation was revealed only after baseline assessment. Blinding of participants and facilitators was not possible because the intervention involved group sessions and the control condition involved less contact. Outcomes were self-reported. During the publication reanalysis, group labels were kept coded until the model specifications had been finalized.

### 2.6. Intervention and Comparator

The program was called “Cultivating Inner Strength: A Growth Support Program for University Students.” It comprised five 90-min group sessions, for a total of 7.5 h. Activities were developed from the motivational and psychological patterns found in the earlier studies. The first author delivered the program with two clinical psychologists experienced in cognitive behavioral work and a clinical social worker experienced in group counseling and family therapy. Table 1 summarizes the sessions.

### 2.7. Outcomes

#### 2.7.1. Primary Outcome

IGD symptoms were assessed with the Chinese version of the GADIS-A (Paschke et al., 2020). The original instrument contains nine symptom items and one duration item. In the doctoral project, the duration item was divided into three indicators of frequency and persistence, yielding a 12-item adapted score that had also been used in the investigators’ earlier work (Zhou et al., 2024b). Higher scores indicate greater symptom severity or risk. At T0, T1, and T2, participants received the same instructions and rated symptoms and consequences with reference to the preceding 12 months; the scoring procedure was unchanged across waves. Because T1 occurred approximately three weeks after T0 and T2 approximately one month later, the reference windows overlapped substantially.

#### 2.7.2. Exploratory Outcome Measures

Exploratory outcomes included average daily internet-use category, K6 psychological distress (Kessler et al., 2002), Rosenberg self-esteem (F. Chen et al., 2015), and brief self-control. Readiness to change problematic internet use was measured with the precontemplation, contemplation, and preparation subscales of the Internet Addiction Improvement Motivation Scale (Park et al., 2012). Four gaming motives from the Gaming Motivation Inventory were examined: introjected regulation, escape, competence, and identity (Király et al., 2022). These motives were selected because they were relevant to the intervention and had appeared in the earlier risk-profile studies (Zhou et al., 2025, 2024b). Higher scores indicate stronger endorsement of the construct.

#### 2.7.3. Intervention Experience

At T1, intervention participants rated perceived self-reflection, control over gaming, awareness of harms, willingness to change, and overall benefit. They also provided free-text comments on each session. These responses were used to describe participants’ experience and to identify parts of the program that might need revision; they were not treated as confirmatory outcomes.

### 2.8. Harms Assessment

Adverse events were not measured with a prespecified instrument. Facilitators invited participants to raise concerns during the sessions, and no serious intervention-related event was reported to the research team. Because harms were not assessed systematically in both groups, the lack of reported events cannot be taken as evidence that no adverse effects occurred.

### 2.9. Patient and Public Involvement

Students were not involved in choosing the research question, designing the trial, or writing the manuscript. Feedback collected after the sessions was used when considering acceptability and future changes to the program.

### 2.10. Statistical Analysis

Baseline characteristics and outcome distributions were summarized by randomized group using means and standard deviations or counts and percentages. Attrition proportions were compared with Fisher’s exact test, and the risk difference and risk ratio were reported with 95% confidence intervals.

The originally planned main between-group analysis was a baseline-adjusted ANCOVA of T1 IGD scores, with T1 score as the dependent variable and randomized group and baseline IGD score as predictors. A corresponding baseline-adjusted T2 model was added during revision. Group-by-baseline interactions were examined to assess homogeneity of regression slopes. As a parsimonious post hoc sensitivity analysis, the T1 and T2 ANCOVA models additionally adjusted for sex. Sex could be linked with certainty for 43 of the 44 completers; the unresolved control record was also alternatively coded as male and female to assess robustness.

A categorical-time linear mixed-effects model was added after data collection to describe trajectories among the 44 participants with complete T0, T1, and T2 data. The model included fixed effects for group, time, and the group-by-time interaction and a random intercept for participant. Post hoc contrasts estimated the between-group differences in change from T0 to T1 and from T0 to T2. Hedges g used the pooled baseline standard deviation, with percentile bootstrap confidence intervals obtained by resampling participants. These mixed-model contrasts and standardized effects are presented as exploratory rather than as prospectively specified co-primary tests.

The same mixed-model specification was applied to the 11 exploratory outcomes. Holm correction was used across the 12 overall interaction tests: IGD and the 11 secondary outcomes. Individual records for the 19 randomized non-completers were unavailable, and no post-randomization outcomes could be recovered for them. An intention-to-treat analysis and a defensible multiple-imputation analysis were therefore not possible. No values were generated by single imputation or last observation carried forward. The original descriptive analyses and ANCOVA were conducted in SPSS 28.0; the publication reanalysis used Python 3.11 and statsmodels 0.14.6. All tests were two-sided, with alpha set at 0.05.

## 3. Results

### 3.1. Participant Flow and Attrition

**Figure 1:**
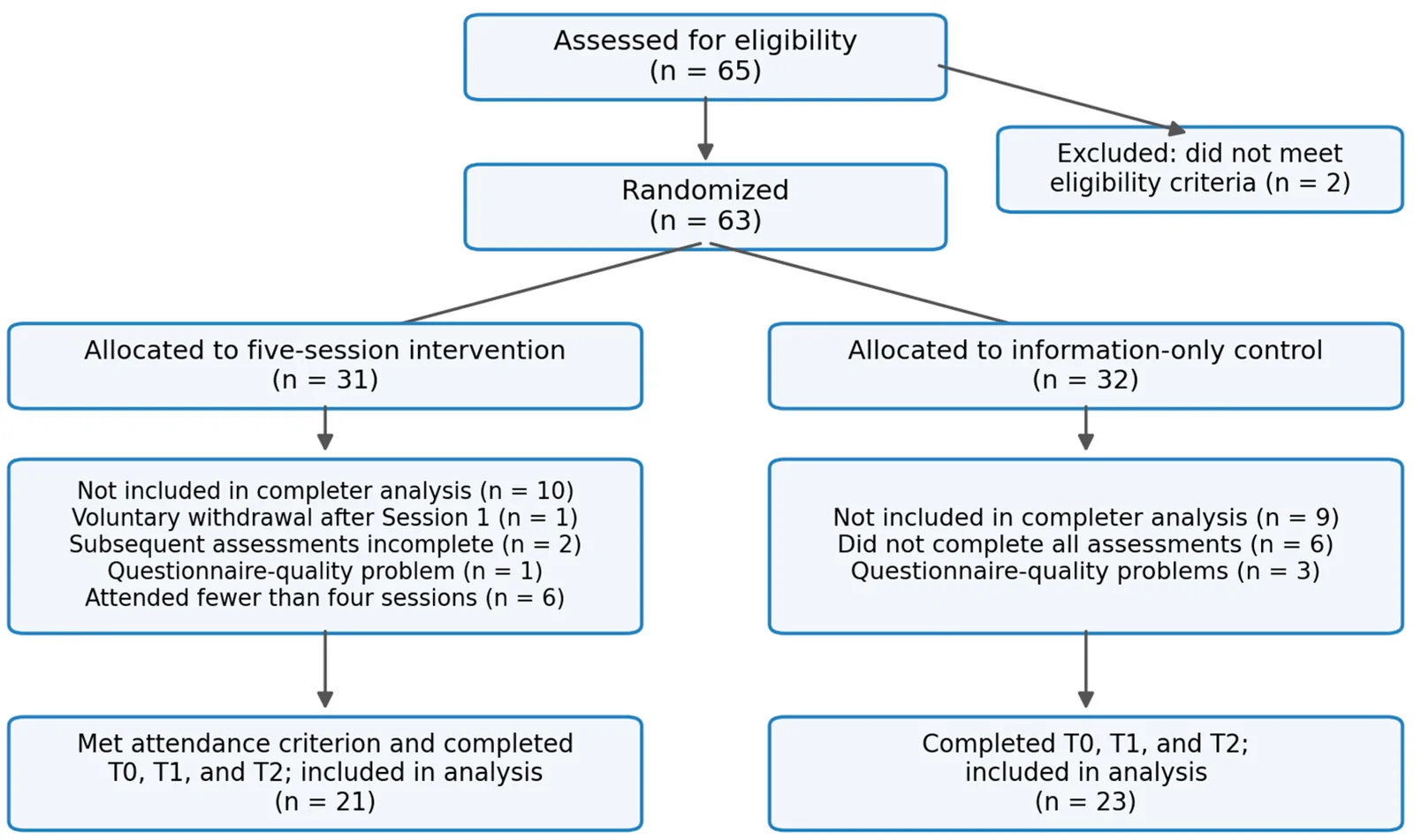
Participant flow by randomized condition. Verified totals and exclusion reasons are shown; post-randomization outcome data were unavailable for the 19 non-completers.

Of 65 students assessed for eligibility, two did not meet the criteria. The remaining 63 were randomized: 31 to the intervention and 32 to the control group. Of the 31 intervention participants, 10 were not included in the completer analysis: one withdrew voluntarily after the first session, two did not complete subsequent assessments, one had questionnaire data that did not meet the quality criterion, and six attended fewer than four sessions. Of the 32 control participants, nine were not included: six did not complete all assessments and three had questionnaire-quality problems. The completer dataset therefore contained 21 intervention participants and 23 controls (Figure 1). The retained records did not permit incomplete assessments to be assigned reliably to T1 versus T2.

Overall attrition was 30.2%: 32.3% in the intervention group and 28.1% in the control group. The risk difference was 4.1 percentage points (95% CI −18.5 to 26.8), the risk ratio was 1.15 (95% CI 0.54 to 2.44), and Fisher’s exact *p* was 0.788. The study did not detect a group difference in attrition, but the estimates were imprecise and selective attrition remains possible.

### 3.2. Baseline Characteristics of the Completer Sample

The 44 completers included 16 men and 28 women and had a mean age of about 19.7 years. Table 2 presents their baseline characteristics. The intervention group included a larger proportion of men and had a mean baseline IGD score 2.12 points higher than the control group.

### 3.3. Intervention and Comparator Delivery

All five sessions were delivered within the planned study period. By definition, the 21 intervention participants in the analytic sample attended at least four sessions and completed all assessments. The control lecture was also delivered as planned. The analytic records did not contain formal fidelity ratings or complete attendance-by-session data for non-completers. Comments were generally favorable for the psychoeducation, self-distancing, cooperative group, and integration sessions; responses to the mindfulness session were mixed. These comments were not compared with the control group.

### 3.4. Primary Outcome: IGD Symptoms

Mean IGD scores in the intervention group were 25.86 (SD 5.09) at T0, 23.00 (SD 5.92) at T1, and 24.19 (SD 4.96) at T2. Control-group means were 23.74 (SD 4.75), 26.04 (SD 5.36), and 23.74 (SD 6.57), respectively. In the post hoc mixed-effects model, the overall group-by-time interaction was significant (likelihood-ratio chi-square(2) = 14.03, *p* < 0.001; Holm-adjusted *p* = 0.0108).

Between T0 and T1, the intervention mean fell by 2.86 points, whereas the control mean rose by 2.30 points. The estimated between-group difference in change was −5.16 points (95% CI −7.81 to −2.51; *p* < 0.001), corresponding to Hedges g = −1.03 (bootstrap 95% CI −1.74 to −0.51). The contrast reflects both the reduction in the intervention group and the temporary increase in the control group; it should not be read as a 5.16-point within-person reduction caused solely by the program.

Between T0 and T2, the intervention mean was 1.67 points below baseline and the control mean was unchanged. The between-group difference in change was −1.67 points (95% CI −4.31 to 0.98; *p* = 0.217), with Hedges g = −0.33 (bootstrap 95% CI −1.01 to 0.33). The data therefore did not show a maintained comparative effect at one month.

In the originally planned T1 ANCOVA, adjusted mean IGD scores were 22.18 for the intervention group and 26.80 for the control group. The adjusted difference was −4.62 points (95% CI −7.33 to −1.91), F(1,41) = 11.88, *p* = 0.0013, partial eta-squared = 0.225. This result was consistent with the immediate post hoc mixed-model contrast.

**Figure 2:**
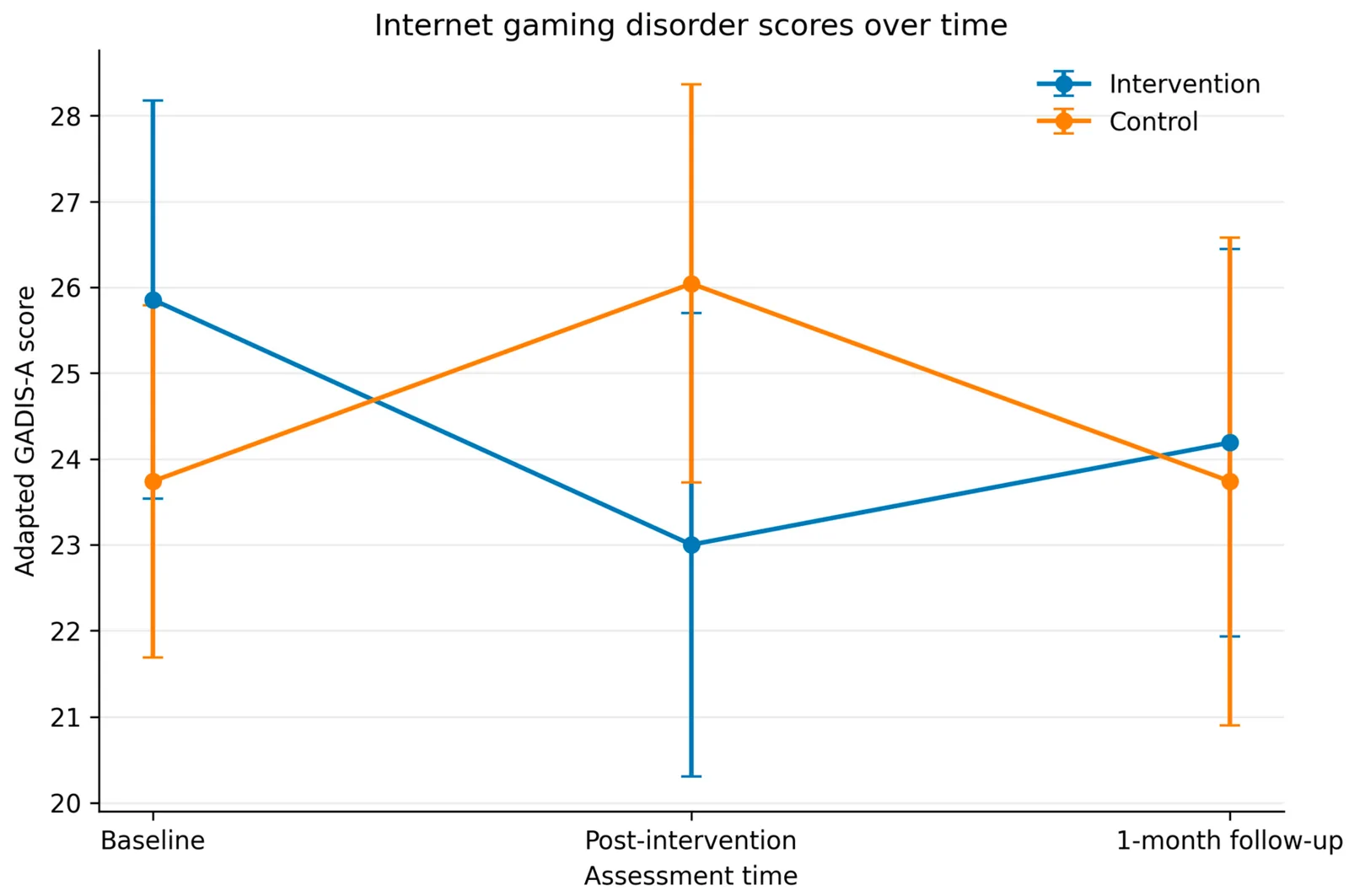
Mean adapted GADIS-A scores by randomized group and assessment time among completers. Error bars are 95% confidence intervals for the group means.

In the added baseline-adjusted T2 ANCOVA, adjusted means were 23.49 and 24.38, and the adjusted difference was −0.89 points (95% CI −4.03 to 2.25), F(1,41) = 0.33, *p* = 0.572, partial eta-squared = 0.008. The group-by-baseline interaction was not significant at T1 (*p* = 0.816) or T2 (*p* = 0.216). In the 43-case models additionally adjusted for sex, the intervention-control difference was −4.47 points at T1 (95% CI −7.23 to −1.71, *p* = 0.002) and −0.49 points at T2 (95% CI −3.70 to 2.71, *p* = 0.758). Alternative coding of the one unresolved control record did not change the substantive interpretation. The model-estimated changes are summarized in Table 3, and the group means over time are shown in Figure 2.

### 3.5. Secondary Exploratory Outcomes

None of the exploratory outcomes showed a significant overall group-by-time interaction. Unadjusted interaction *p* values ranged from 0.157 to 0.948, and all Holm-adjusted *p* values for these outcomes were 1.000. Thus, the trial found no between-group evidence of change in daily internet use, psychological distress, self-esteem, self-control, readiness to change, or the selected gaming motives. Within-group changes were not interpreted as treatment effects. The corresponding mixed-model estimates are presented in Table 4.

### 3.6. Harms

No serious adverse event was reported to the research team. Adverse events were not assessed systematically in both groups, so incidence could not be compared.

## 4. Discussion

### 4.1. Principal Findings

Among the 44 participants who completed all three assessments, the originally planned baseline-adjusted T1 ANCOVA and the post hoc mixed-effects model both favored the intervention immediately after treatment. At one month, however, the baseline-adjusted and mixed-model between-group differences were smaller and not statistically significant. Adjustment for sex did not materially change this interpretation. None of the exploratory psychological or motivational outcomes showed a group-by-time interaction after correction for multiple testing. The findings therefore support a preliminary immediate difference among selected completers, but not a maintained effect or an intention-to-treat estimate for all randomized participants.

The post-intervention contrast reflected a 2.86-point reduction in the intervention group and a 2.30-point increase in the control group. The control mean returned to baseline at follow-up. Temporary fluctuation, assessment reactivity, events during the study period, regression to the mean, or chance may have contributed to this pattern. The estimated difference of −5.16 points is a comparison of group trajectories, not the amount by which the program reduced each participant’s score. Its large standardized effect is also uncertain because it was estimated from a small completer sample and was not sustained at follow-up.

### 4.2. Interpretation in Light of Recent Treatment Evidence

The GADIS-A retained the same preceding-12-month instructions at all three assessments, preserving scoring comparability across waves but producing substantially overlapping reference windows. The immediate score difference therefore should not be interpreted as onset or remission of a 12-month disorder within three weeks. At the same time, repeated standardized symptom measures are commonly used in intervention studies and may register changes in participants’ current appraisal of ongoing symptoms. The finding is best treated as a short-term symptom-severity signal, with less temporal specificity for accumulated functional consequences.

Recent reviews suggest that psychological treatment can reduce gaming-disorder symptoms, but they also describe a literature marked by small samples, heterogeneous interventions, inconsistent diagnostic methods, and substantial risk of bias. A 2023 review of randomized trials found benefits for several psychological approaches but rated much of the evidence as having medium or high risk of bias (Y. Chen et al., 2023). Danielsen et al. (2024) reported a moderate pooled effect across 38 intervention studies, together with small-study effects, possible publication bias, and limited standardization. Two 2025 meta-analyses likewise found overall symptom improvement, particularly for psychotherapy and cognitive behavioral approaches, while emphasizing considerable heterogeneity in treatment content, comparison groups, outcome definitions, and study quality (Harpas et al., 2025; Ock et al., 2025).

At the brief end of the international literature, the four-session PROTECT trial showed that a school-based group program could change symptom trajectories among at-risk adolescents, although it addressed indicated prevention rather than treatment and used substantially longer follow-up (Lindenberg et al., 2022). Ji and Wong’s (2023) eight-session trial provides the closest contextual comparison because it tested an integrated motivational cognitive-behavioral group intervention in Chinese secondary vocational-school students using intention-to-treat analyses and three- and six-month follow-ups. The present study addresses a narrower implementation question in adult tertiary vocational-college students: whether a five-session format can be delivered within a campus timetable and show an immediate signal that merits a larger trial. The current one-month findings do not establish maintenance.

The immediate effect in this trial was larger than most pooled estimates, but direct comparison would be misleading. Our estimate came from one college, a small completer sample, and a control group whose mean score increased temporarily after baseline. The lack of a significant group difference at one month is consistent with the uneven follow-up findings in the wider literature. On the present evidence, the program appears capable of producing short-term change in some students who complete it; sustained benefit remains unproven.

### 4.3. Mindfulness, Treatment Dose, and the Absence of Maintained Effects

Two recent mindfulness trials provide useful context. Ni et al. (2024) randomized 64 adults with IGD to eight sessions of mindfulness meditation or progressive muscle relaxation. Mindfulness produced greater reductions in IGD severity and craving, and change in craving was associated with altered frontopallidal activity, although longer-term maintenance was not examined. Yoon et al. (2026) evaluated eight sessions of mindfulness-based cognitive therapy in 46 college-aged students. Relative to a no-treatment control, participants improved in IGD symptoms, perceived stress, and self-control, with most differences still present four weeks later. Both interventions delivered substantially more mindfulness practice than the single dedicated mindfulness session in our program, and their populations and comparison conditions differed from ours.

In the present program, mindfulness was one component of a broader five-session package, and participant reactions to that session were mixed. A single session, without structured home practice or booster contact, may have been insufficient to produce stable changes in attention or emotion regulation. The absence of between-group differences in self-control and other proposed process measures is consistent with that interpretation. The intervention group also had considerably more contact with facilitators and peers than the information-only control group. Attention, expectancy, group cohesion, and social support may therefore have contributed to the immediate result. A longer intervention, guided practice between sessions, booster contacts, and a contact-matched active control would allow a more specific test.

### 4.4. Mechanisms, Secondary Outcomes, and the Intervention Model

Motivation and self-regulation remain plausible targets for intervention. Recent longitudinal findings suggest that self-control dimensions and gaming motives explain more variation in gaming disorder than game genre or platform, with escape, coping, and competence-related motives among the relevant factors (Cudo et al., 2023). Our earlier studies also linked low self-control and maladaptive motives to IGD and identified different combinations of motivational and psychological vulnerability (Zhou et al., 2025, 2024b). These associations helped to shape the program, but risk predictors do not automatically become mechanisms of treatment change.

Self-control, self-esteem, readiness to change, and the measured gaming motives did not change differently between groups. This trial therefore provides no evidence that those variables mediated the immediate IGD difference. Other processes may have mattered, including craving, cue reactivity, self-monitoring, treatment expectancy, social support, or temporary changes in daily routine. Future trials should identify a small number of candidate mediators in advance and measure them during treatment. Repeated assessments of craving, urges, and coping may be more sensitive to a brief intervention than trait-like measures collected only at the main assessment points.

### 4.5. Attrition, Strengths, and Limitations

Attrition is the main threat to interpretation. Nineteen of the 63 randomized participants were not included in the completer analysis. In the intervention condition, exclusions followed withdrawal, incomplete follow-up, a questionnaire-quality problem, or attendance at fewer than four sessions; in the control condition, they followed incomplete assessments or questionnaire-quality problems. Because inclusion depended on post-randomization behavior and was not defined identically across conditions, the original protection of randomization was not fully preserved. Similar attrition proportions in the two groups do not exclude bias. Because individual records for non-completers were unavailable, they could not be compared with completers and an intention-to-treat analysis could not be performed. The estimates therefore describe selected completers, not everyone who was randomized.

The trial nevertheless had several useful features: random allocation, a clear empirical rationale for the intervention content, three assessment points, direct comparison of group trajectories, effect estimates with confidence intervals, and correction for multiple exploratory tests. The limitations are substantial. Registration occurred after enrollment began, and the mixed-model analysis was specified after data collection. The intervention completers began with a higher mean IGD score and included more men; baseline and sex-adjusted analyses reduced but could not eliminate concern about imbalance, regression to the mean, or unmeasured selection. The same 12-month GADIS-A reference period was retained across closely spaced assessments, which preserved comparability but reduced temporal specificity. The initial recruitment target of 80 was not reached because the college teaching calendar and fixed intervention window limited further enrollment; this may have reduced precision for smaller secondary and exploratory effects. The sample was small and drawn from one vocational college. Eligibility was based on a symptom threshold rather than a clinical diagnosis, and the GADIS-A scoring was adapted. Participants and facilitators were not blinded, outcomes were self-reported, and the control condition was not matched for contact time. Follow-up lasted only one month. Treatment fidelity, concurrent care, adverse events, and exposure to individual program components were not monitored systematically. Because several approaches were combined, the immediate difference cannot be attributed to motivation work, mindfulness, cognitive behavioral content, group processes, or any single component. Data were collected in 2024; subsequent changes in games, platforms, campus routines, and help-seeking practices should be considered when generalizing the findings. Although this interval may affect the contemporaneity of the findings, it does not, by itself, alter the internal comparison between the randomized groups.

### 4.6. Implications for Research and Practice

A subsequent trial should register the protocol and analysis plan before recruitment, recruit across several universities, and retain baseline and follow-up records for every randomized participant, including those who stop attending sessions. Assessments at three and six months would provide a more informative test of maintenance. A multi-timescale measurement strategy would also be useful: a validated 12-month measure could be retained for diagnostic continuity, while shorter past-week or past-month measures of impaired control, craving, gaming time, and functional interference could capture proximal change. Reminders, make-up sessions, structured between-session practice, and booster contacts may improve retention. Prospective measures of craving, cue reactivity, momentary self-regulation, treatment expectancy, alliance, attendance, and component exposure would help clarify how change occurs. Treatment fidelity and adverse events should also be recorded. A contact-matched comparator would reduce uncertainty about nonspecific attention effects, and later dismantling or factorial designs could identify the components that are actually needed. Until such evidence is available, this program should be regarded as a campus-based intervention prototype rather than an established treatment.

## 5. Conclusions

Among students who completed the study, the five-session program was associated with a large immediate between-group difference in IGD symptom change. The difference was not evident one month later, and the exploratory psychological and motivational outcomes did not differ between groups. Retrospective registration, post hoc analysis, 30.2% attrition, and the absence of intention-to-treat data limit the conclusion. These preliminary outcomes among selected completers support further testing of the campus-based intervention in a larger, prospectively registered trial with longer follow-up and complete outcome collection.

## Figures and Tables

**Table 1:** Content and intended targets of the five-session intervention.

Session	Target	Core Activities	Planned Dose
1. Psychoeducation	Problem awareness, virtual identity, readiness to change	Case discussion, video examples, reflection on internet dependence and identity in virtual environments, group discussion, brief meditation	90 min
2. “No-I” writing	Self-distancing and reduced dependence on external evaluation	Describe a recent day in the third person, discuss intrinsic value and self-evaluation, brief meditation	90 min
3. Group counseling: Human Knot	Real-world competence, cooperation, group cohesion	Cooperative problem-solving activity followed by reflection on process, contribution, and competence	90 min
4. Mindfulness	Attentional pause, emotion regulation, deliberate choice	Posture and breathing instruction, focused-attention breathing, discussion of mindful pauses in everyday gaming-related situations	90 min
5. Integration and planning	Consolidation and behavioral planning	Film and TED-talk material, group discussion, self-management plan, identification of alternative sources of connection and achievement	90 min

The control group attended one information-only lecture on internet dependence and IGD, equivalent to the lecture component of Session 1, and received no further therapeutic contact. The control condition was not matched for contact time. Both groups continued their usual academic and campus activities. Apart from excluding students who were already receiving psychiatric treatment, the study did not systematically record concurrent psychological care.

**Table 2:** Baseline characteristics of participants included in the completer analysis. Values are mean (SD) unless stated otherwise.

Characteristic	Intervention (*n* = 21)	Control (*n* = 23)
Age, years	19.67 (0.73)	19.65 (1.23)
Men, *n* (%)	10 (47.6)	6 (26.1)
Women, *n* (%)	11 (52.4)	17 (73.9)
Daily internet use	4.05 (1.47)	4.13 (1.01)
IGD score	25.86 (5.09)	23.74 (4.75)
K6 psychological distress	16.81 (4.87)	18.35 (5.53)
Self-esteem	26.19 (3.27)	25.04 (4.11)
Self-control	37.33 (4.98)	36.57 (5.62)
Precontemplation	10.33 (1.83)	10.30 (3.38)
Contemplation	13.48 (1.50)	13.78 (3.01)
Preparation	17.67 (2.44)	18.00 (4.75)
Introjected regulation	4.03 (1.35)	3.81 (1.28)
Escape motive	4.76 (1.14)	5.07 (1.09)
Competence motive	4.98 (1.33)	4.72 (1.37)
Identity motive	4.43 (1.20)	4.27 (1.36)

**Table 3:** Model-estimated changes in IGD among 44 completers. Negative differences favor the intervention group.

Time Contrast	Intervention Change	Control Change	Difference in Change (95% CI)	*p*	Hedges g (95% CI)
T0 to T1	−2.86	+2.30	−5.16 (−7.81 to −2.51)	<0.001	−1.03 (−1.74 to −0.51)
T0 to T2	−1.67	0.00	−1.67 (−4.31 to 0.98)	0.217	−0.33 (−1.01 to 0.33)

**Table 4:** Post hoc mixed-model results for exploratory outcomes. Difference in change is intervention minus control; no overall interaction was statistically significant.

Outcome	Overall Group-by-Time *p*	T0–T1 Difference in Change (95% CI; *p*)	T0–T2 Difference in Change (95% CI; *p*)	Holm *p*
Daily internet use	0.380	0.59 (−0.24 to 1.41; 0.163)	0.33 (−0.50 to 1.15; 0.437)	1.000
K6 distress	0.213	0.05 (−3.64 to 3.74; 0.978)	2.92 (−0.77 to 6.61; 0.121)	1.000
Self-esteem	0.901	−0.32 (−2.53 to 1.89; 0.775)	−0.51 (−2.72 to 1.70; 0.652)	1.000
Self-control	0.948	0.46 (−3.06 to 3.98; 0.797)	−0.08 (−3.61 to 3.44; 0.963)	1.000
Precontemplation	0.814	−0.01 (−1.84 to 1.83; 0.995)	−0.52 (−2.36 to 1.31; 0.576)	1.000
Contemplation	0.646	0.68 (−1.08 to 2.44; 0.448)	0.77 (−0.99 to 2.53; 0.392)	1.000
Preparation	0.429	1.65 (−0.83 to 4.13; 0.191)	0.80 (−1.68 to 3.27; 0.530)	1.000
Introjected regulation	0.686	−0.32 (−1.06 to 0.42; 0.401)	−0.23 (−0.98 to 0.51; 0.536)	1.000
Escape motive	0.421	0.19 (−0.39 to 0.77; 0.518)	−0.20 (−0.78 to 0.38; 0.500)	1.000
Competence motive	0.157	−0.46 (−1.14 to 0.21; 0.178)	−0.65 (−1.33 to 0.03; 0.059)	1.000
Identity motive	0.586	−0.15 (−0.86 to 0.57; 0.689)	−0.37 (−1.09 to 0.34; 0.303)	1.000

## Data Availability

The de-identified data and analysis code supporting this study are provided in the Supplementary Materials.

## Supplementary Materials

The following supporting information can be downloaded at https://www.mdpi.com/article/10.3390/bs16091500/s1: Supplementary File, additional analysis details and aggregate statistical output tables; statistical analysis code; and a de-identified participant-level dataset for the 44 completers.

## References

[B1-behavsci-16-01500] American Psychiatric Association (2013). Diagnostic and statistical manual of mental disorders.

[B2-behavsci-16-01500] Balhara Y. P. S., Sarkar S., Laspal N., Bhargava R., Yadav Z. (2023). A randomized controlled trial to assess effectiveness of GamE—An e-health intervention to self-manage gaming with an aim to prevent gaming disorder. Asian Journal of Psychiatry.

[B3-behavsci-16-01500] Ballabio M., Griffiths M. D., Urbán R., Quartiroli A., Demetrovics Z., Király O. (2017). Do gaming motives mediate between psychiatric symptoms and problematic gaming? An empirical survey study. Addiction Research & Theory.

[B4-behavsci-16-01500] Bäcklund C., Elbe P., Gavelin H. M., Sörman D. E., Ljungberg J. K. (2022). Gaming motivations and gaming disorder symptoms: A systematic review and meta-analysis. Journal of Behavioral Addictions.

[B5-behavsci-16-01500] Brand M., Wegmann E., Stark R., Müller A., Wölfling K., Robbins T. W., Potenza M. N. (2019). The interaction of Person-Affect-Cognition-Execution (I-PACE) model for addictive behaviors: Update, generalization to addictive behaviors beyond internet-use disorders, and specification of the process character of addictive behaviors. Neuroscience & Biobehavioral Reviews.

[B6-behavsci-16-01500] Chen F., Bi C., Han M. (2015). The reliability and validity of the Chinese version of the revised-positive version of Rosenberg self-esteem scale. Advances in Psychology.

[B7-behavsci-16-01500] Chen Y., Lu J., Wang L., Gao X. (2023). Effective interventions for gaming disorder: A systematic review of randomized control trials. Frontiers in Psychiatry.

[B8-behavsci-16-01500] China Internet Network Information Center (2022). The 50th statistical report on China’s internet development.

[B9-behavsci-16-01500] Cohen J. (1988). Statistical power analysis for the behavioral sciences.

[B10-behavsci-16-01500] Cudo A., Kopiś-Posiej N., Griffiths M. D. (2023). The role of self-control dimensions, game motivation, game genre, and game platforms in gaming disorder: Cross-sectional and longitudinal findings. Psychology Research and Behavior Management.

[B11-behavsci-16-01500] Danielsen P. A., Mentzoni R. A., Låg T. (2024). Treatment effects of therapeutic interventions for gaming disorder: A systematic review and meta-analysis. Addictive Behaviors.

[B12-behavsci-16-01500] Deng L.-Y., Liu L., Xia C.-C., Lan J., Zhang J.-T., Fang X.-Y. (2017). Craving behavior intervention in ameliorating college students’ internet game disorder: A longitudinal study. Frontiers in Psychology.

[B13-behavsci-16-01500] Fam J. Y. (2018). Prevalence of internet gaming disorder in adolescents: A meta-analysis across three decades. Scandinavian Journal of Psychology.

[B14-behavsci-16-01500] Gu X., Mao E. Z. (2023). The impacts of academic stress on college students’ problematic smartphone use and internet gaming disorder under the background of neijuan: Hierarchical regressions with mediational analysis on escape and coping motives. Frontiers in Psychiatry.

[B15-behavsci-16-01500] Harpas I., Stevens M., Radunz M., Williamson P., Hamamura T., Svendsen O., King D. L. (2025). Treatment of gaming disorder: A systematic review and meta-analysis. Psychiatry Research.

[B16-behavsci-16-01500] Hopewell S., Chan A.-W., Collins G. S., Hróbjartsson A., Moher D., Schulz K. F., Tunn R., Aggarwal R., Berkwits M., Berlin J. A., Bhandari N., Butcher N. J., Campbell M. K., Chidebe R. C. W., Elbourne D., Farmer A., Fergusson D. A., Golub R. M., Goodman S. N., Boutron I. (2025). CONSORT 2025 statement: Updated guideline for reporting randomized trials. JAMA.

[B17-behavsci-16-01500] Ji Y., Wong D. F. K. (2023). Effectiveness of an integrated motivational cognitive-behavioral group intervention for adolescents with gaming disorder: A randomized controlled trial. Addiction.

[B18-behavsci-16-01500] Kessler R. C., Andrews G., Colpe L. J., Hiripi E., Mroczek D. K., Normand S.-L. T., Walters E. E., Zaslavsky A. M. (2002). Short screening scales to monitor population prevalences and trends in non-specific psychological distress. Psychological Medicine.

[B19-behavsci-16-01500] King D. L., Delfabbro P. H., Wu A. M. S., Doh Y. Y., Kuss D. J., Pallesen S., Mentzoni R., Carragher N., Sakuma H. (2017). Treatment of internet gaming disorder: An international systematic review and CONSORT evaluation. Clinical Psychology Review.

[B20-behavsci-16-01500] Király O., Billieux J., King D. L., Urbán R., Koncz P., Polgár E., Demetrovics Z. (2022). A comprehensive model to understand and assess the motivational background of video game use: The Gaming Motivation Inventory (GMI). Journal of Behavioral Addictions.

[B21-behavsci-16-01500] Lei X. Y., Xiao L. M., Liu Y. N., Li Y. M. (2016). Prevalence of depression among Chinese university students: A meta-analysis. PLoS ONE.

[B22-behavsci-16-01500] Li W., Garland E. L., McGovern P., O’Brien J. E., Tronnier C., Howard M. O. (2017). Mindfulness-oriented recovery enhancement for internet gaming disorder in U.S. adults: A stage I randomized controlled trial. Psychology of Addictive Behaviors.

[B23-behavsci-16-01500] Lindenberg K., Kindt S., Szász-Janocha C. (2022). Effectiveness of cognitive behavioral therapy-based intervention in preventing gaming disorder and unspecified internet use disorder in adolescents: A cluster randomized clinical trial. JAMA Network Open.

[B24-behavsci-16-01500] Long J., Liu T., Liu Y., Hao W., Maurage P., Billieux J. (2018). Prevalence and correlates of problematic online gaming: A systematic review of the evidence published in Chinese. Current Addiction Reports.

[B25-behavsci-16-01500] Lu P., Qiu J., Huang S., Wang X., Han S., Zhu S., Ning Y., Zeng F.-F., Yuan Y. (2025). Interventions for digital addiction: Umbrella review of meta-analyses. Journal of Medical Internet Research.

[B26-behavsci-16-01500] Mihara S., Higuchi S. (2017). Cross-sectional and longitudinal epidemiological studies of internet gaming disorder: A systematic review of the literature. Psychiatry and Clinical Neurosciences.

[B27-behavsci-16-01500] Ni H., Wang H., Ma X., Li S., Liu C., Song X., Potenza M. N., Dong G.-H. (2024). Efficacy and neural mechanisms of mindfulness meditation among adults with internet gaming disorder: A randomized clinical trial. JAMA Network Open.

[B28-behavsci-16-01500] Ock C. M., Lee H. S., Chae J., Kim H. (2025). Effectiveness of non-pharmacological interventions on gaming disorder: A systematic review and meta-analysis. Psychiatry Investigation.

[B29-behavsci-16-01500] Park J. W., Park K. H., Lee I. J., Kwon M., Kim D. J. (2012). Standardization study of internet addiction improvement motivation scale. Psychiatry Investigation.

[B30-behavsci-16-01500] Paschke K., Austermann M. I., Thomasius R. (2020). Assessing ICD-11 gaming disorder in adolescent gamers: Development and validation of the Gaming Disorder Scale for Adolescents (GADIS-A). Journal of Clinical Medicine.

[B31-behavsci-16-01500] Petry N. M., O’Brien C. P. (2013). Internet gaming disorder and the DSM-5. Addiction.

[B32-behavsci-16-01500] Pontes H. M., Schivinski B., Sindermann C., Li M., Becker B., Zhou M., Montag C. (2021). Measurement and conceptualization of gaming disorder according to the World Health Organization framework: The development of the gaming disorder test. International Journal of Mental Health and Addiction.

[B33-behavsci-16-01500] Poon E. C. S., Soh X. C., Poh T. J. H., Tan A. C. S., Hartanto A. (2026). Global prevalence of internet gaming disorder: An umbrella review of meta-analytic evidence and implications for child and adolescent psychiatry. Brain Sciences.

[B34-behavsci-16-01500] Reed G. M., First M. B., Kogan C. S., Hyman S. E., Gureje O., Gaebel W., Maj M., Stein D. J., Maercker A., Tyrer P., Claudino A., Garralda E., Salvador-Carulla L., Ray R., Saunders J. B., Dua T., Poznyak V., Medina-Mora M. E., Pike K. M., Saxena S. (2019). Innovations and changes in the ICD-11 classification of mental, behavioural and neurodevelopmental disorders. World Psychiatry.

[B35-behavsci-16-01500] Stevens M. W. R., King D. L., Dorstyn D., Delfabbro P. H. (2019). Cognitive-behavioral therapy for internet gaming disorder: A systematic review and meta-analysis. Clinical Psychology & Psychotherapy.

[B36-behavsci-16-01500] Su W., Fang X., Miller J. K., Wang Y. (2011). Internet-based intervention for the treatment of online addiction for college students in China: A pilot study of the Healthy Online Self-Helping Center. Cyberpsychology, Behavior, and Social Networking.

[B37-behavsci-16-01500] Wölfling K., Müller K. W., Dreier M., Ruckes C., Deuster O., Batra A., Mann K., Musalek M., Schuster A., Lemenager T., Hanke S., Beutel M. E. (2019). Efficacy of short-term treatment of internet and computer game addiction: A randomized clinical trial. JAMA Psychiatry.

[B38-behavsci-16-01500] Yao Y.-W., Chen P.-R., Li C. R., Hare T. A., Li S., Zhang J.-T., Liu L., Ma S.-S., Fang X.-Y. (2017). Combined reality therapy and mindfulness meditation decrease intertemporal decisional impulsivity in young adults with internet gaming disorder. Computers in Human Behavior.

[B39-behavsci-16-01500] Yoon A., Park Y., Kim M. A., Chiu I.-M., Solomon P., Kim S. J., Oh H., Kuyken W. (2026). A pilot study of a mindfulness-based cognitive therapy for internet gaming disorder among college-aged students in South Korea. Mindfulness.

[B40-behavsci-16-01500] Zajac K., Ginley M. K., Chang R., Petry N. M. (2017). Treatments for internet gaming disorder and internet addiction: A systematic review. Psychology of Addictive Behaviors.

[B41-behavsci-16-01500] Zhou R., Morita N., Ogai Y., Saito T., Zhang X., Ogawa M., Yang W. (2025). Predictors and patterns of internet gaming disorder using a regression tree based on game motivation and psychological factors. Minerva Psychiatry.

[B42-behavsci-16-01500] Zhou R., Morita N., Ogai Y., Saito T., Zhang X., Yang W., Yang F. (2024a). Meta-analysis of internet gaming disorder prevalence: Assessing the impacts of DSM-5 and ICD-11 diagnostic criteria. International Journal of Environmental Research and Public Health.

[B43-behavsci-16-01500] Zhou R., Morita N., Zhu C., Ogai Y., Saito T., Yang W., Ogawa M., Zhang H. (2024b). The relationship between self-control and internet gaming disorder and problematic social networking site use: The mediation effects of internet use motives. Frontiers in Psychiatry.

