# Supplementary material for: Preliminary Outcomes of a Five-Session Motivation-Centered Program for Internet Gaming Disorder: A Completer Analysis of a Randomized Trial
Source: Behav Sci (Basel). 2026 Aug 26;16(9):1500. doi: 10.3390/bs16091500 (PMC13603776; doi:10.3390/bs16091500)
Supplement: Supplementary file 1 [file behavsci-16-01500-s001.zip › Supplementary_Analysis_Details.pdf]

Supplementary File S1. Additional Analysis Details and Statistical Output Tables

Short-Term Effects of a Five-Session Motivation-Centered Group Program on Internet Gaming Disorder Symptoms in Chinese University Students: A Randomized Controlled Trial

S1. Analysis population and data structure

The analyses used the 44 participants with complete observations at baseline (T0), post-intervention (T1), and one-month follow-up (T2): 21 in the intervention group and 23 in the control group. Participants were analyzed according to their randomized group. No outcome values were imputed. Individual-level records for the 19 randomized non-completers were not available in the retained dataset, so the analyses are completer analyses rather than a full intention-to-treat analysis.

The supplied data file is in wide format, with one row per participant. The analysis script reshapes the data to one record per participant, outcome, and time point. Group is coded 1 for intervention and 2 for control. The variable named InternetTime records the six-category response to the questionnaire item on average daily online gaming time.

S2. Mixed-effects models and multiplicity

For each outcome, time was entered as a categorical factor. Fixed effects were group, time, and the group-by-time interaction, with a random intercept for participant. Models were fitted by maximum likelihood. The overall interaction was tested by a likelihood-ratio comparison with a model that omitted the interaction. Planned contrasts estimated the between-group difference in change from T0 to T1 and from T0 to T2.

For IGD, Hedges g was calculated for the difference in change using the pooled baseline standard deviation. Percentile bootstrap confidence intervals were obtained from 10,000 participant-level resamples using seed 20260805. Holm correction was applied across the 12 overall interaction tests: IGD and the 11 exploratory outcomes.

S3. Primary IGD analysis

The overall group-by-time interaction for IGD was significant, likelihood-ratio chi-square(2) = 14.03, $p < 0.001$; Holm-adjusted $p = 0.0108$.

Table S1. Between-group differences in IGD change among the 44 completers.

Contrast	Intervention change	Control change	Difference in change (95% CI)	p	Hedges g (95% CI)
T0 to T1	-2.86	+2.30	-5.16 (-7.81 to -2.51)	<0.001	-1.03 (-1.75 to -0.50)
T0 to T2	-1.67	+0.00	-1.67 (-4.31 to +0.98)	0.217	-0.33 (-1.02 to +0.29)

Note. Difference in change is intervention minus control. Negative values favor the intervention group for IGD.

S4. Exploratory outcomes

No exploratory outcome showed a statistically significant overall group-by-time interaction. All Holm-adjusted p values were 1.000.

Table S2. Mixed-model results for the exploratory outcomes.

Outcome	Overall interaction p	T0-T1 difference in change (95% CI; p)	T0-T2 difference in change (95% CI; p)	Holm p
Daily online gaming time category	0.380	+0.59 (-0.24 to +1.41; p=0.163)	+0.33 (-0.50 to +1.15; p=0.437)	1.000
K6 distress	0.213	+0.05 (-3.64 to +3.74; p=0.978)	+2.92 (-0.77 to +6.61; p=0.121)	1.000
Self-esteem	0.901	-0.32 (-2.53 to +1.89; p=0.775)	-0.51 (-2.72 to +1.70; p=0.652)	1.000
Self-control	0.948	+0.46 (-3.06 to +3.98; p=0.797)	-0.08 (-3.61 to +3.44; p=0.963)	1.000
Precontemplation	0.814	-0.01 (-1.84 to +1.83; p=0.995)	-0.52 (-2.36 to +1.31; p=0.576)	1.000
Contemplation	0.646	+0.68 (-1.08 to +2.44; p=0.448)	+0.77 (-0.99 to +2.53; p=0.392)	1.000
Preparation	0.429	+1.65 (-0.83 to +4.13; p=0.191)	+0.80 (-1.68 to +3.27; p=0.530)	1.000
Introjected regulation	0.686	-0.32 (-1.06 to +0.42; p=0.401)	-0.23 (-0.98 to +0.51; p=0.536)	1.000
Escape motive	0.421	+0.19 (-0.39 to +0.77; p=0.518)	-0.20 (-0.78 to +0.38; p=0.500)	1.000
Competence motive	0.157	-0.46 (-1.14 to +0.21; p=0.178)	-0.65 (-1.33 to +0.03; p=0.059)	1.000
Identity motive	0.586	-0.15 (-0.86 to +0.57; p=0.689)	-0.37 (-1.09 to +0.34; p=0.303)	1.000

Note. Difference in change is intervention minus control. The global interaction p value is unadjusted; Holm p is adjusted across 12 outcomes.

S5. Post-intervention ANCOVA sensitivity analysis

A baseline-adjusted ANCOVA of T1 IGD scores was retained as a sensitivity analysis because it corresponded to the original doctoral analysis. The adjusted mean was 22.18 in the intervention group and 26.80 in the control group. The adjusted difference (intervention minus control) was -4.62 points (95% CI -7.33 to -1.91), $F(1,41) = 11.88$, $p = 0.0013$, partial eta-squared = 0.225.

Table S3. ANCOVA diagnostic checks.

Diagnostic	p
Baseline-by-group interaction (homogeneity of slopes)	0.816
Shapiro-Wilk test of residual normality	0.758
Breusch-Pagan test of heteroscedasticity	0.077

S6. Attrition

Attrition was compared between randomized groups to describe whether the proportion excluded from the completer analysis differed materially by allocation.

Table S4. Participant retention by randomized group.

Group	Randomized	Included in analysis	Not included	Attrition rate
Intervention	31	21	10	32.3%
Control	32	23	9	28.1%
Total	63	44	19	30.2%

The intervention-minus-control risk difference was 4.1 percentage points (95% CI -18.5 to 26.8). The risk ratio was 1.15 (95% CI 0.54 to 2.44), and Fisher's exact p was 0.788. Similar attrition proportions do not exclude selective attrition.

S7. Reproducibility files

The supplementary package contains: (1) this document; (2) a de-identified participant-level dataset for the 44 completers; and (3) a Python script that reproduces data reshaping, descriptive summaries, mixed-effects models, the ANCOVA sensitivity analysis, the attrition comparison, bootstrap confidence intervals, and figure generation. The script requires NumPy, pandas, SciPy, and statsmodels. It does not create an intention-to-treat analysis.
